# Mental Disorders, Cognitive Impairment and the Risk of Suicide in Older Adults

**DOI:** 10.3389/fpsyt.2021.695286

**Published:** 2021-08-25

**Authors:** Agnieszka Kułak-Bejda, Grzegorz Bejda, Napoleon Waszkiewicz

**Affiliations:** ^1^Department of Psychiatry, Medical University of Bialystok, Bialystok, Poland; ^2^The School of Medical Science in Bialystok, Bialystok, Poland

**Keywords:** suicide, elderly, cognitive impairment, dementia, depression, bipolar disorder, anxiety

## Abstract

More than 600 million people are aged 60 years and over are living in the world. The World Health Organization estimates that this number will double by 2025 to 2 billion older people. Suicide among people over the age of 60 is one of the most acute problems. The factors strongly associated with suicide are mentioned: physical illnesses, such as cancer, neurologic disorder, pain, liver disease, genital disorders, or rheumatoid disorders. Moreover, neurologic conditions, especially stroke, may affect decision-making processes, cognitive capacity, and language deficit. In addition to dementia, the most common mental disorders are mood and anxiety disorders. A common symptom of these disorders in the elderly is cognitive impairment. This study aimed to present the relationship between cognitive impairment due to dementia, mood disorders and anxiety, and an increased risk of suicide among older people. Dementia is a disease where the risk of suicide is significant. Many studies demonstrated that older adults with dementia had an increased risk of suicide death than those without dementia. Similar conclusions apply to prodromal dementia Depression is also a disease with a high risk of suicide. Many researchers found that a higher level of depression was associated with suicide attempts and suicide ideation. Bipolar disorder is the second entity in mood disorders with an increased risk of suicide among the elderly. Apart from suicidal thoughts, bipolar disorder is characterized by high mortality. In the group of anxiety disorders, the most significant risk of suicide occurs when depression is present. In turn, suicide thoughts are more common in social phobia than in other anxiety disorders. Suicide among the elderly is a serious public health problem. There is a positive correlation between mental disorders such as dementia, depression, bipolar disorder, or anxiety and the prevalence of suicide in the elderly. Therefore, the elderly should be comprehensively provided with psychiatric and psychological support.

## Introduction

The World Health Organization (WHO) reported that more than 600 million people age 60 years and older worldwide. The WHO further estimated that, by 2025, this number would double to two billion older people (1). It is well-known that older people are at greater risk for diseases and body injuries, poverty, social isolation, loneliness, and loss of independence, all of which contribute to deterioration in mental health (2, 3). The prevalence rate of suicide ranges from 8.54 to 33% (3–5). In older adults, the most frequently diagnosed mental disorders are anxiety disorders (10.9%) and mood disorders such as depression (7.4%) (3, 6). Moreover, suicide is a global concern. Almost 78% of all completed suicides occur in low- and middle-income countries, and in general, suicides account for 1.4% of premature deaths worldwide (7).

The rate of suicide has been shown to increase with advancing age, and one of the most acute problems is suicide among people over the age of 60. Older men and women have been identified as having the highest suicide rate in almost every country, reaching 48.7/100,000 in the US for white men (more than four times that nation's age-adjusted rate of 11.1/100,000) and 140/100,000 in rural China for men (6, 8).

Among the factors strongly associated with suicide are physical illnesses, such as cancer, neurologic disorder, pain, liver disease, genital disorders, and rheumatoid disorders. Moreover, neurologic conditions, especially stroke, may affect decision-making processes, cognitive capacity, and language (9, 10). These risk factors, combined with a lack of social connection and a sense of meaninglessness, contribute to the occurrence of suicidal behavior (9–11).

In older adults, suicide is often a consequence of mental disorders such as depression, anxiety disorder, insomnia, Alzheimer's disease, and vascular dementia (12). All these disease entities are also strongly associated with cognitive impairment.

Cognitive appraisal theory is a theory of emotions. It states that a person's evaluative judgment (or appraisal) of a situation, event, or object determines or contributes to their emotional response to it. Cognitive appraisal theory is based on the James-Lange theory of emotions and considers that a given physiological response can give rise to various emotional responses. The theory was initially proposed by American psychologist Stanley Schachter in 1964 and has later been developed further by other researchers. In an experiment carried out by Schachter and Singer, participants were injected with adrenaline and not told that this would cause a heightened level of physiological arousal. When placed in situations designed to elicit either anger or euphoria, these subjects would attribute their state of arousal to their problem (13).

Cognitive impairment plays a role in attempted suicide among older people. The literature mentions the following risk factors: dysfunctional cognitive control, executive function, and problem-solving. The loss of these abilities makes it difficult to cope with life problems functionally and, thus, increases the risk of suicide (14).

Older people newly diagnosed with dementia are significantly more at risk for suicide than their peers without dementia. Individuals diagnosed with dementia had a 54% increased risk for suicide within the 1st year after diagnosis. The risk was exceptionally high among those aged 74 years and younger (12).

Several studies have considered cognitive impairment in late-life depression both qualitatively and quantitatively. Studies that included a clinical diagnosis of major depression, a comprehensive assessment of cognition, and a healthy comparison group have documented deficits in episodic memory, speed of information processing, executive functioning, and visual-spatial ability (15).

Deficits in the speed of information processing and executive functioning might be particularly pertinent. Three recent studies reported that slowed rate of information processing or working memory deficits appears to mediate the cognitive impairment associated with depression predominantly. Mechanisms that might increase the risk depression poses for developing Alzheimer's disease (16). Late-life depression is associated with both chronic elevations of adrenal glucocorticoid production and cerebrovascular disease. Together, these factors may lead to hippocampal atrophy and generalized ischemia (17).

Generalized ischemia often has a preference for frontostriatal regions, leading to abnormalities that could also serve to maintain or cause subsequent depressive episodes. These factors can also lower brain or cognitive reserve. When other pre-existing Alzheimer's disease casual risk factors are present, this can hasten the progression of underlying Alzheimer's disease pathology to clinical manifestation of this disease entity (17).

Some reports show depressive symptoms are linked to suicide in patients with cognitive impairment (12, 18–21). Little is yet known about the relationship between cognitive impairment itself and suicide.

Worse cognitive functioning is associated with more frequent suicidal ideas in those individuals with depression even when depression severity was taken into account (22).

This study aimed to present the relationship between cognitive impairment due to dementia, mood disorders and anxiety, and an increased risk of suicide among older adults. The authors provide an overview of the literature from the main databases (Medline, Web of Science, EMBASE, and the Cochrane Database of Systematic Reviews) from the latest years.

## Dementia

Dementia is a disease entity characterized by progressive cognitive impairment and behavioral changes. Dementia is a general term used to refer to diseases including Alzheimer's disease, vascular dementia, Lewy body dementia, frontotemporal dementia, and mixed dementia (23, 24). Data have shown that about 50% of all dementia is related to Alzheimer's disease (24, 25). Generally, patients with dementia present the following symptoms: progressive impairments in memory, thinking, and behavior, which have a strong negative influence on everyday activities. Other common symptoms include difficulties with language, decreased motivation, and emotional problems, such as depression, psychotic hallucinations, delusions, apathy, anxiety, or aggression (26).

Tu et al. analyzed data from the Taiwan National Health Insurance Research Database and compared 1,189 patients age ≥ 65 who attempted suicide and 4,756 age- and sex-matched control subjects. The methods used by suicide attempters included drug overdose (13.5%), hanging or drowning (4.1%), physical injury (44.2%), and poison (38.5%). The researchers also found that the elderly who attempted suicide had a significantly (*p* < 0.001) higher likelihood of developing dementia than controls. Another conclusion they reached is that demographic geriatric suicide attempts were significantly associated with an elevated risk of developing dementia (hazard ratio [HR]: 7.40, 95% CI: 6.11–8.97, *p* < 0.001) in later life (27).

Annor et al. analyzed data from 2013 to 2016 from the Georgia Alzheimer's Disease and Related Dementia registry that were linked with data from the Georgia Vital Records and Georgia Violent Death Reporting System. They identified 91 residents with dementia who died by suicide. The suicide rate among persons with dementia was 9.3 per 100,000 person-years overall and higher among those diagnosed in the past 12 months (424.5/100,000 person-years). They concluded that male gender, the onset of dementia before age 65, and a recent diagnosis of dementia are predictive factors of suicide (28).

Zucca et al. determined the prevalence of suicidal ideation and attempts in patients with behavioral frontotemporal dementia (bvFTD) by assessing possible risk factors for suicide. They found that patients with bvFTD who attempted suicide showed a higher global Scale for Suicide Ideation (SSI) score in comparison to those who presented suicidal ideation alone (*P* < 0.001). Patients with bvFTD were found to have significantly higher functional and cognitive impairment levels and were more apathetic and impulsive. However, no differences in neuropsychological characteristics were identified when comparing patients with bvFTD at risk for suicide and patients with bvFTD with no suicide risk (29).

Lai et al. analyzed data from two databases, the US Department of Veterans Affairs (VA) National Patient Care Database and the Department of Veterans Affairs (VA) National Suicide Prevention Applications Network (SPAN), and estimated the 2-year prevalence of mental health disorders across five dementia subtypes during fiscal years 2012 and 2013. They found that patients with FTD had the highest 2-year prevalence of psychiatric disorders and suicidal behavior (nearly 4% having suicidal ideation and 0.5% having a suicidal plan or having attempted suicide). Likewise, patients with VD showed a high 2-year prevalence of suicidal behavior (3%) and other psychiatric disorders such as anxiety (14%) and substance use (17%) (30).

Choi et al. used the National Health Insurance Service Senior Cohort data. They included 36,541 older adults with newly diagnosed dementia such as Alzheimer's disease, vascular dementia, and other/unspecified dementia from 2004 to 2012. They attempted to estimate suicide risk within 1 year of dementia diagnosis. They verified 46 deaths by suicide during the 1st year after a dementia diagnosis. Older adults with dementia had an increased risk of death by suicide than those without dementia (31).

Koyama et al. examined 634 patients with dementia and observed suicidal ideation in 10%. These results correlated with the severity of behavioral and psychological dementia symptoms (32).

Richard-Devantoy et al. suggested that poor cognitive control may contribute to high suicide rates in old age. They also demonstrated that cognitive control deficits in older patients with any high-lethality suicide attempt might undermine their ability to solve real-life problems, precipitating a catastrophic accumulation of stressors (33).

By contrast, a link has been identified between suicide and prodromal dementia. Gujral et al. compared 67 non-suicidal depressed older adults, 63 depressed suicide ideators, 44 late-onset and 48 early-onset suicide attempters, and 56 non-psychiatric comparison groups. They found that both attempter groups presented worse executive functioning than the non-suicidal depressed group. Moreover, poorer global cognition and processing speed were demonstrated by late-onset attempters compared to non-suicidal depressed older adults. They also showed more flawed memory than early-onset attempters (34).

## Mood Disorders

Depressive disorders are described in the Diagnostic and Statistical Manual for Mental Disorders (5th edition; DSM-5). Among the group of mood disorders, the DSM-5 includes major depressive disorder (MDD), persistent depressive disorder (dysthymia), bipolar I and II disorder (BD), cyclothymic disorder, and premenstrual dysphoric disorder (35).

The prevalence of the major depressive disorder in adults age 65 and older ranges from 1–5% in the community. Furthermore, significant depressive symptoms are present in approximately 15% of older adults (36).

Lee and Atteraya analyzed data from the Survey of Living Conditions and Welfare Needs of Korean Older Persons, which included 10,279 persons. They found that people between 65 and 69 years old have suicide ideation more often than those 80 years and older. A higher level of depression (OR = 1.19) was strong associated with suicide ideation. In addition, poverty (OR = 1.59) and exposure to abuse (OR = 2.37) were associated with suicide ideation (37).

Nakamura et al. analyzed data from 63,026 men and 72,268 women age 65 years and older. They showed that the male suicide standardized mortality ratio (SMR) was positively correlated with depressive symptoms (*p* = 0.002). The female suicide SMR was positively correlated with the rate of depressive symptoms (ρ = 0.258) (38).

Choi et al. focused on a specific group of patients. They assessed suicide risk in the elderly within 1 year after stroke and attempted to correlate it with depression. Patients with depression had an increased risk of suicide after stroke (AHR = 2.9; 95% CI, 1.8–4.8), and this applied to post-stroke depression as well (AHR = 4.1; 95% CI, 1.8–9.5) (31). However, compared to stroke patients without depression, both pre-and post-stroke depression suicidality was higher (AHR = 4.8; 95% CI, 2.1–11.1) (39).

For their study, Kiosses et al. recruited 74 older participants (65–95 years old) with MDD and cognitive impairment. They found that the Montgomery–Aasberg Depression Rating Scale's negative emotions scores were significantly associated with suicidal ideation during treatment (F_[1, 165]_ = 12.73, *p* = 0.0005) (40).

Yeh et al. analyzed data from national health insurance databases in Taiwan. They showed that the risk of suicide was higher in those who had depression (OR = 3.49, 95% CI = 2.2–5.4) and bipolar disorder (OR = 1.98, 95% CI = 1.1–3.6) (41).

Tan et al. attempted to identify the differences between two age groups, middle-aged (45–64 years) and older-aged people (65+ years), who had self-harmed. They found that the 65+ age group had a history of depression (*p* < 0.0001) and had been diagnosed with depression at the time of their attempt (*p* < 0.0001). Moreover, suicidal intent was more common among older-aged people who had self-harmed (*p* = 0.004), and this group had lower survival rates in the 12 months following their self-harm attempt (risk ratio = 7.5; 95% CI = 3.1–18.1) (42).

Woo et al. characterized the high-intent suicide attempters admitted to emergency departments. They demonstrated that the number of older than 66 years increased as the strength of suicidal intent increased (*P* = 0.04). They also had higher Hamilton Depression Rating Scale (HDRS) scores, higher premeditation rates, and sustained suicidal ideation (43).

Park et al. analyzed the data from the Survey of Living Conditions and Welfare Needs of Korean Older Persons. The prevalences of depression and suicidal ideation were 30.3 and 11.2%, respectively. Moreover, they found that 60.5% of suicidal ideation could be prevented or reduced if depression was eliminated (44).

The 220 older adults with bipolar disorder (BD) recruited to the study conducted by O'Rourke et al. found that older adults with BD who reported low satisfaction with life and current depressive symptoms and misused alcohol also reported having significantly higher levels of suicidal ideation (45). The same authors attempted to describe predictors of suicidal ideation among older adults with BD. They confirmed that depressive symptoms and cognitive failures could be predictors of suicidal ideation (46).

Almeida et al. analyzed data from 37,768 men ages 65–85 years with bipolar disorder. They showed that BD was also associated with increased mortality (HR = 1.51, 95% CI 1.28–1.77). Suicide was one of the most common causes of death in this group of patients (47).

Belvederi Murri et al. drew interesting conclusions from their study. They found that cognitive impairments linked with BD were associated with an increased likelihood of disability and recent aggressive behavior but not suicidal thoughts (48).

## Anxiety Disorders

Generalized anxiety disorder (GAD) is a state of chronic, uncontrolled anxiety. This anxiety disrupts daily functioning and causes sleep disturbance. In turn, panic disorder is characterized by a recurrent, unexpected attack of intense anxiety. It causes physical and cognitive dysfunctions and impairs everyday functioning. By contrast, a specific phobia is an exaggerated fear of a particular object or situation (49, 50).

According to Pary et al., fear of falling occurs in about 50% of older persons who have fallen recently (49).

Bendixen et al. included 218 older adults in their study. They measured the severity of anxiety symptoms using the Geriatric Anxiety Inventory (GAI) and the Montgomery–Aasberg Depression Rating Scale (MADRS) to assess the severity of depression. They showed that higher GAI scores were significantly associated with suicidality (β = 0.206, *p* = 0.006). Moreover, higher GAI scores were related to scores on the MADRS (β = 0.233, *p* = 0.002) (51).

Petkus et al. examined age differences in death and suicidal ideation in patients with anxiety disorders. Interestingly, the authors showed that older participants with social anxiety disorder were significantly more likely to report thoughts of death and suicidal thoughts than older adults with other anxiety disorders (52).

## Discussion

Suicide is problem that affects older adults. In the USA, suicide rates are high among older men, with men ages 85 and older having the highest rate of any other group. Moreover, suicide attempts by the elderly are much more likely to end in death than among young people. The following factors influence this fact: older adults use more deadly methods, are less often found and saved, and they are less likely to recover from complications due to age and comorbidities (9, 11, 53).

In many studies, the relationship between disability, pain, physical illnesses (like cancer), and suicidal behavior in older adults has been demonstrated (9, 10, 54, 55).

Moreover, the systematic review conducted by Fässberg et al. showed that completed suicide specifically had been associated with psychiatric disorders (e.g., major depressive disorder), addictions, and sleep disturbances (54). Among other risk factors, the researchers mentioned marital status (widowed or divorced), family conflicts, material problems, and previous suicide attempts (55, 56).

Among the prisoners' specific study group, suicide rates are higher than those in the same age group in the general population. The authors associate these findings with the lack of proper diagnosis of mental disorders and their specific treatment (56).

Szanto et al. found three pathways to suicidal behavior in older adults, and the authors distinguished: very high levels of cognitive and dispositional risk factors, dysfunctional personality traits, and impulsive decision-making, and cognitive deficits (57).

Interestingly, Jung et al. showed that depression and suicidal ideation levels in the religiously affiliated group were not significantly different from that of the religiously non-affiliated group. Moreover, there were no significant differences between Christians and Buddhists (58).

Kiosses et al. presented psychosocial intervention to reduce suicide risk in older adults who have been hospitalized after a suicide attempt or with suicidal ideation. Cognitive Reappraisal Intervention for Suicide Prevention (CRISP) assumed: an emotional crisis precedes hospitalization for suicidality and patients have had difficulty dealing with this emotional crisis; this emotional crisis is related to personalized triggers; identifying these personalized triggers and the associated negative emotions and providing strategies for adaptive response and improvement of suicide prevention. The authors expect that CRISP may reduce suicide risk at the post-discharge period, where the patients are particularly vulnerable to suicide (40).

In turn, Kim et al. analyzed the effectiveness of a community-based program for suicide prevention among elders with early-stage dementia. They found that the developed suicide prevention program significantly affected the perceived health status, social support, depression, and suicidal ideation of elders with early-stage dementia (59).

Sakashita and Oyama concluded that community interventions are essential in reducing suicide in older adults. Furthermore, integrating universal, selective, and indicated prevention strategies might be necessary for this process. Perhaps the most important relationship is between selective and indicated preventive interventions (60).

The International Research Group for Suicide Among the Elderly proposes that multifaceted strategies should improve resilience and positive aging and involve family and community guardians. Also, telecommunications should be expanded to reach vulnerable older people and assess the impact of resource constraints. Moreover, physicians of all specialties should be educated on suicide by older people and actively participate in preventive programs (61).

Older people are most vulnerable to health problems due to various factors: depression, anxiety, dementia, neurocognitive disorders, social isolation, loss of relatives, pain, and chronic physical diseases. These mentioned factors increase the risk for suicidal behavior. So, these risk factors should be taken into account in suicide prevention strategies (9).

Our results have several medical implications. Family doctors should be involved in the prevention of suicidal behavior in older people. They are the first to have contact with older suicidal people. Older adults have a higher prevalence of physical disorders and more often visit their doctors (62).

Furthermore, older adults had more frequent contact with the family doctor than with the psychiatric services before suicide. Additionally, anxiety disorder and depression are associated with suicide, and the treatment of these psychiatric conditions should be enhanced in older adults (63).

The loss of a spouse, grief, hopelessness increase the risk of suicidal thoughts or behaviors. Older adults who have been widowed, socially isolated, and who have cognitive problems should increase our attention in health consultations (64).

## Strengths and Limitations

This literature review presents the most recent findings on dementia, mood disorders, anxiety, and suicidal behaviors in older adults. However, some limitations should be highlighted. First, the most significant part of the studies has been performed on small population samples with clinical heterogeneity according to age and the disease stage. Second, very few studies explored the link between suicide and the different types of dementia. Third, we included the literature from recent years.

## Conclusions

Suicide among people over the age of 60 is a frequent problem. Older men and women have been identified as having the highest suicide rate in almost every country. Dementia is a disease where the risk of suicide is significant. Older adults with dementia had an increased risk of suicide death compared to those without dementia. Also, prodromal dementia, depression, and bipolar disorder are diseases with a high risk of suicide.

Furthermore, bipolar disorder is characterized by high mortality. Families, social support systems, and health care providers should learn about suicide in older people. And they should discuss suicide warning signs and how to provide support. It is essential to make physicians who have contact with seniors aware of the possible occurrence of suicidal thoughts, especially if mental disorders accompany them. We suggest that family doctors should be able to perform suicide risk tests among seniors.

## Author Contributions

AK-B contributed to the conception and design of the study and wrote the first draft of the manuscript. AK-B and GB analyzed the database. NW supervised the preparation of the final version of the manuscript. All authors contributed to the revision of the manuscript and read and approved the presented version.

## Conflict of Interest

The authors declare that the research was conducted in the absence of any commercial or financial relationships that could be construed as a potential conflict of interest.

## Publisher's Note

All claims expressed in this article are solely those of the authors and do not necessarily represent those of their affiliated organizations, or those of the publisher, the editors and the reviewers. Any product that may be evaluated in this article, or claim that may be made by its manufacturer, is not guaranteed or endorsed by the publisher.
